# Kindlin-2 regulates hepatic stellate cells activation and liver fibrogenesis

**DOI:** 10.1038/s41420-018-0095-9

**Published:** 2018-09-12

**Authors:** Jun Yu, Yinan Hu, Yi Gao, Qinghai Li, Zhilin Zeng, Yong Li, Huilong Chen

**Affiliations:** 1 Department of Thoracic Surgery, Tongji Hospital of Tongji Medical College, Huazhong University of Science and Technology, Wuhan, China; 2 Department of Respiratory and Critical Care Medicine, Tongji Hospital, Tongji Medical College, Huazhong University of Science and Technology, Wuhan, China; 3 Hepatobiliary and Pancreas Diagnosis and Treatment Center, Shiyan Taihe Hospital, Hubei University of Medicine, Shiyan, Hubei China; 4 Department of Infectious Disease, Tongji Hospital of Tongji Medical College, Huazhong University of Science and Technology, Wuhan, China

**Keywords:** Liver fibrosis, Liver cirrhosis

## Abstract

Liver fibrosis, the common response associated with chronic liver diseases, ultimately leads to cirrhosis, a major public health problem worldwide. Activation of hepatic stellate cells (HSCs) by transforming growth factor (TGF)-β1 is a key step in liver fibrosis. Here we report that Kindlin-2 expression is elevated in the livers of mice with experimental liver fibrosis and also in the livers of patients with liver fibrosis. TGF-β1 increases Kindlin-2 expression in cultured HSCs in a p38 and ERK mitogen-activated protein kinase (MAPK)-dependent manner, partly. More importantly, Kindlin-2 deficiency significantly attenuated mouse liver fibrosis and HSC activation. Mechanistically, Kindlin-2 promotes TGF-β signaling through upregulation of Smad2 and Smad3 phosphorylation. Our work demonstrates an important role for Kindlin-2 in liver fibrosis, and inhibiting Kindlin-2 in the livers may represent a novel strategy to treat liver fibrosis.

## Introduction

Liver fibrosis is a tightly controlled wound healing response to virtually all forms of chronic liver injury due to viral infections, metabolic, and autoimmune diseases, which results in excessive accumulation of extracellular matrix (ECM) and impaired normal liver function^1^. It is widely accepted that activated hepatic stellate cells (HSCs) play a pivotal role during development of liver fibrosis^2^. In response to liver injury, quiescent HSCs activate and differentiate into myofibroblast-like cells that are fibrogenic, contractile, and proliferative^2,3^. Transforming growth factor (TGF)-β1 has been identified as the most potent mediator of HSC activation in liver fibrosis among several growth factors and cytokines^4^. TGF-β1 signals through the canonical Smad signaling pathway involving TGF-β receptor-mediated phosphorylation of Smad2 and Smad3 (p-Smad2/3), which form complexes with Smad4 and translocate to the nucleus to regulate gene transcription^5,6^. In addition to the Smad pathway, TGF-β1 can also activate noncanonical Smad pathways, which including MAP kinase (MAPK) pathways, Rho-like GTPase signaling pathways, and phosphatidylinositol-3-kinase (PI3K)/AKT pathways^7^. Both canonical and noncanonical pathways have been linked to HSC activation and liver fibrosis^8^.

Kindlins consist of three members, Kindlin-1, -2, and -3, which are a family of adapter proteins that mediate cell–cell and cell–matrix adhesions^9–11^. Kindlin-2, also known as Mig-2, encoded by *FERMT2* gene, is widely expressed and evolutionarily conserved^12^. Kindlin-2 has emerged as a key regulator of integrin activation, which lead to actin remodeling, cell migration, and adhesion^13,14^. Global deficiency of Kindlin-2 in mice caused early embryonic lethality, suggesting that Kindlin-2 plays a vital role in development^12^. Furthermore, recent reports have demonstrated that Kindlin-2 participates in numerous biological and pathological processes, such as cell apoptosis, differentiation, survival, and carcinogenesis^15–20^. Recently, Kindlin-2 was also reported to play an essential role in fibrotic disorders. Wei and co-workers demonstrated that Kindlin-2 is highly expressed in human and mouse fibrotic kidney, and depletion of Kindlin-2 attenuates experimental renal fibrosis^21^; on the contrary, knockout of Kindlin-2 expression in cardiac myocyte or depletion of Kindlin-2 resulted in progressive cardiac fibrosis^22,23^. Currently, the effect of Kindlin-2 in liver fibrosis remains unknown.

In the present study, we conducted a series of experiments to clarify the role of Kindlin-2 in the pathogenesis of liver fibrosis. We showed that Kindlin-2 is upregulated in human and mouse fibrotic livers, and this upregulation is mediated by TGF-β1 through noncanonical Smad pathways in HSC, and then enhances HSC activation. Mechanistically, we demonstrated that Kindlin-2 mediates TGF-β signaling through phosphorylation of Smad2/3. Notably, depletion of Kindlin-2 dramatically inactivates the TGF-β/Smad pathway and thereby prevents TGF-β1-induced HSC activation and experimental liver fibrosis. Our study has uncovered an important role of Kindlin-2 in liver fibrosis progression and suggests that inhibition of Kindlin-2 may represent a novel therapeutic approach for the treatment of hepatic fibrosis.

## Results

### Kindlin-2 expression is upregulated in human and mouse liver fibrosis

**Fig. 1:**
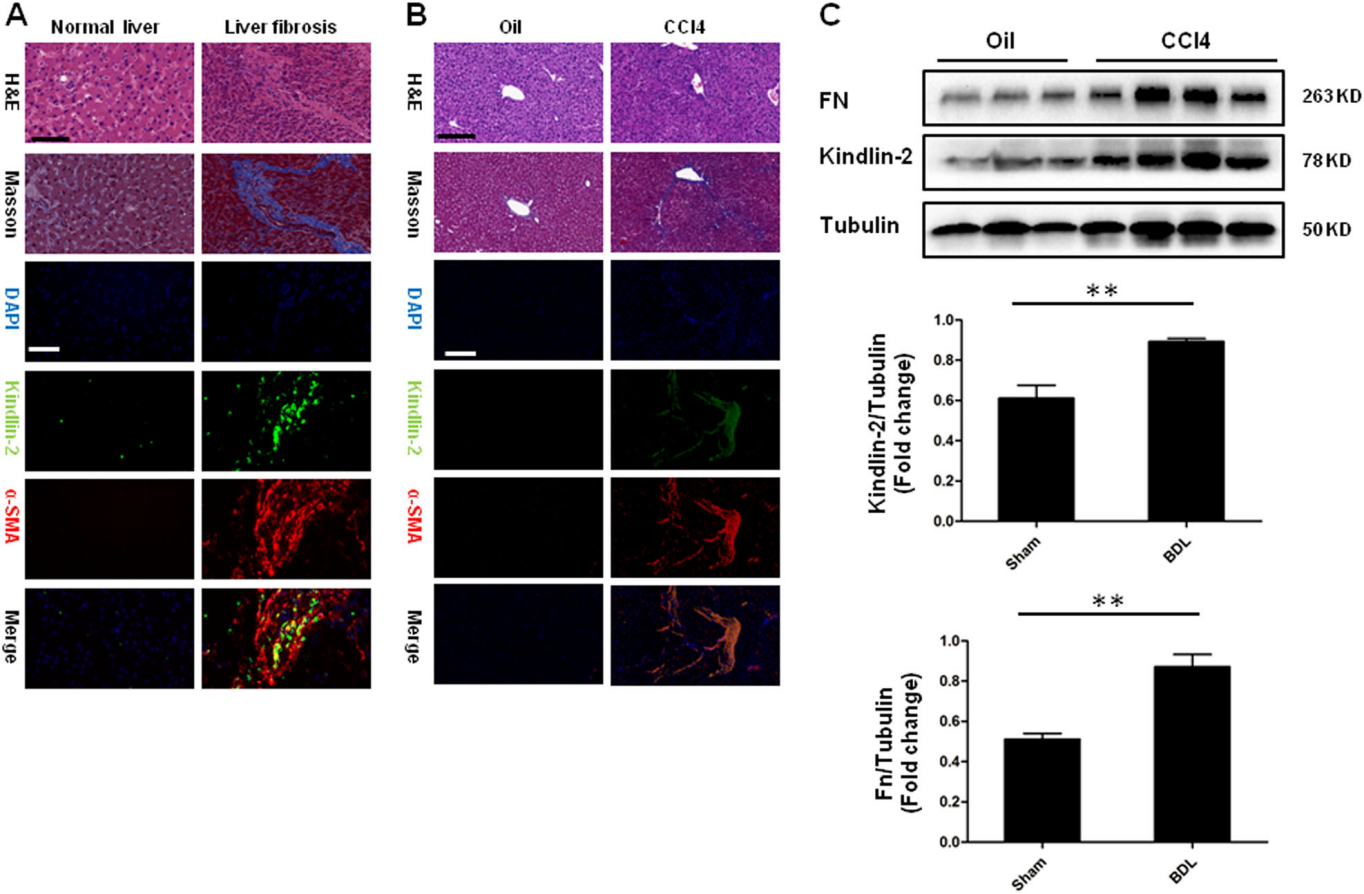
Expression of Kindlin-2 is upregulated in human and mouse liver fibrosis. **a** HE, Masson staining and double immunofluorescence staining for Kindlin-2 (green) and a-SMA (red) in normal (*n* = 5) and fibrotic human livers (*n* = 8). Scale bars = 200 μm. **b** Dual immunofluorescence was used to identify the expression of Kindlin-2 (green) and a-SMA (red) in liver biopsy samples from WT mice with or without injection of CCl_4_ (*n* = 6 for WT and *n* = 6 for CCl_4_). Scale bars = 200 μm. **c** Representative western blot analysis of Kindlin-2 in mouse livers from WT mice with or without injection of CCl_4_. Quantitative analyses of Kindlin-2 and Fn are shown in the lower panel. **p < 0.01

To understand the role of Kindlin-2 in the progression of liver fibrosis, we initially assessed Kindlin-2 levels in human fibrotic livers. As shown in Fig. 1a, using immunofluorescence assay, we found that Kindlin-2 was highly expressed in human liver fibrosis. Double staining with α-SMA showed that activated HSCs express significant high level of Kindlin-2. We then detected the Kindlin-2 expression in mouse fibrotic livers that were induced by intraperitoneal CCl_4_ injection, a well-studied mouse liver fibrosis model^24^. We also observed elevated Kindlin-2 expression in experimental liver fibrosis. Immunofluorescence co-staining Kindlin-2 and α-SMA highlighted a similar expression pattern in mouse as in human fibrotic livers with strongly increased Kindlin-2 levels in activated HSC (Fig. 1b). Moreover, western blot showed that the CCl_4_-induced mouse livers exhibited significant upregulation of Kindlin-2 compared with the control livers (Fig. 1c). The expression of tubulin was not changed upon CCl_4_ treatment. These data demonstrated that increased Kindlin-2 may serve as a critical marker of HSC activation.

### TGF-β upregulates Kindlin-2 expression in HSC via p38 and ERK

**Fig. 2:**
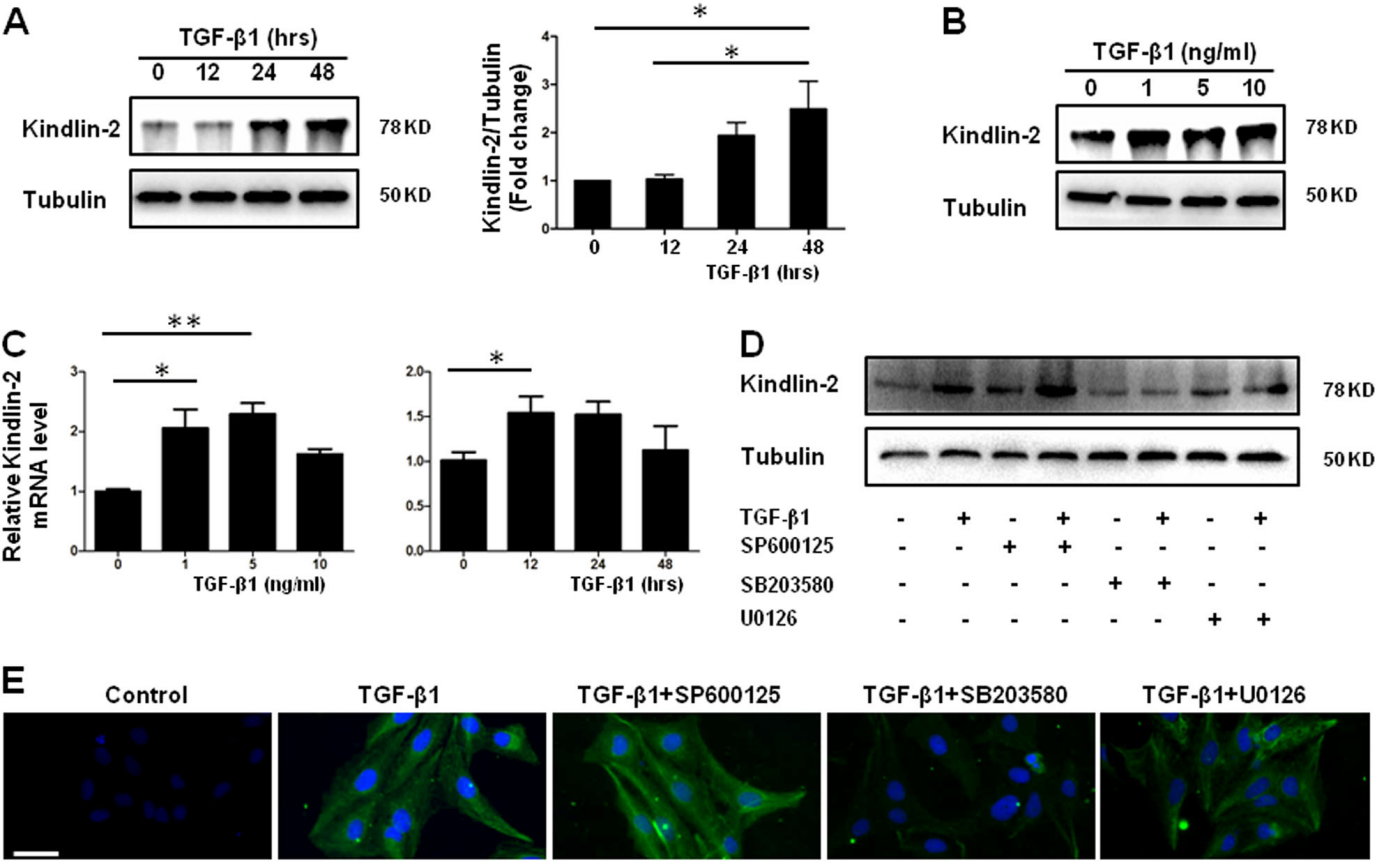
TGF-β1 increases Kindlin-2 expression in HSCs via p38 and ERK MAPK. **a** Representative western blot analysis of Kindlin-2 from LX-2 cells treated with TGF-β1 (10 ng/ml) for the indicated time period. The right panel shows quantitative analyses of Kindlin-2 after normalization with tubulin. **b** Representative western blot analysis of Kindlin-2 from LX-2 cells treated with TGF-β1 for 24 h for indicated concentrations. **c** Quantitative PCR analyses of Kindlin-2 mRNA from LX-2 cells treated with indicated concentrations of TGF-β1 for 24 h (left panel) or 10 ng/ml of TGF-β1 for indicated time intervals (right panel). Western blot (**d**) and immunofluorescence analysis (**e**) of Kindlin-2 from LX-2 cells were treated with or without SP600125 (10 μM), U0126 (1 μM), or SB203580 (1 μM), following TGF-β1 treatment for 24 h. The data are representative of three independent experiments. Scale bars = 200 μm. *p < 0.05. **p < 0.01

We next examined Kindlin-2 levels in LX-2 cells^25^, a well-established human HSC cell line upon TGF-β treatment. As illustrated in Fig 2a, b, TGF-β1 increases Kindlin-2 protein levels in a time-dependent and dose-dependent manner. In addition, elevated Kindlin-2 mRNA level was also observed in the whole RNA extracted from TGF-β1-treated LX-2 cells (Fig. 2c). To investigate how TGF-β1 triggers Kindlin-2 expression, TGF-β1 signaling inhibitors were added before TGF-β1 treatment. We observed that p38 MAPK inhibitor SB203580 and extracellular signal-regulated kinase (ERK) inhibitor U0126, but not c-Jun N-terminal kinase (JNK) inhibitor SP600125, blocked the increase effect of TGF-β1 on Kindlin-2 expression in LX-2 cells (Fig. 2d, e). Taken together, we demonstrated that the TGF-β1 triggers Kindlin-2 expression via p38 and ERK MAPK pathway.

### Kindlin-2 promotes TGF-β-induced HSC activation

**Fig. 3:**
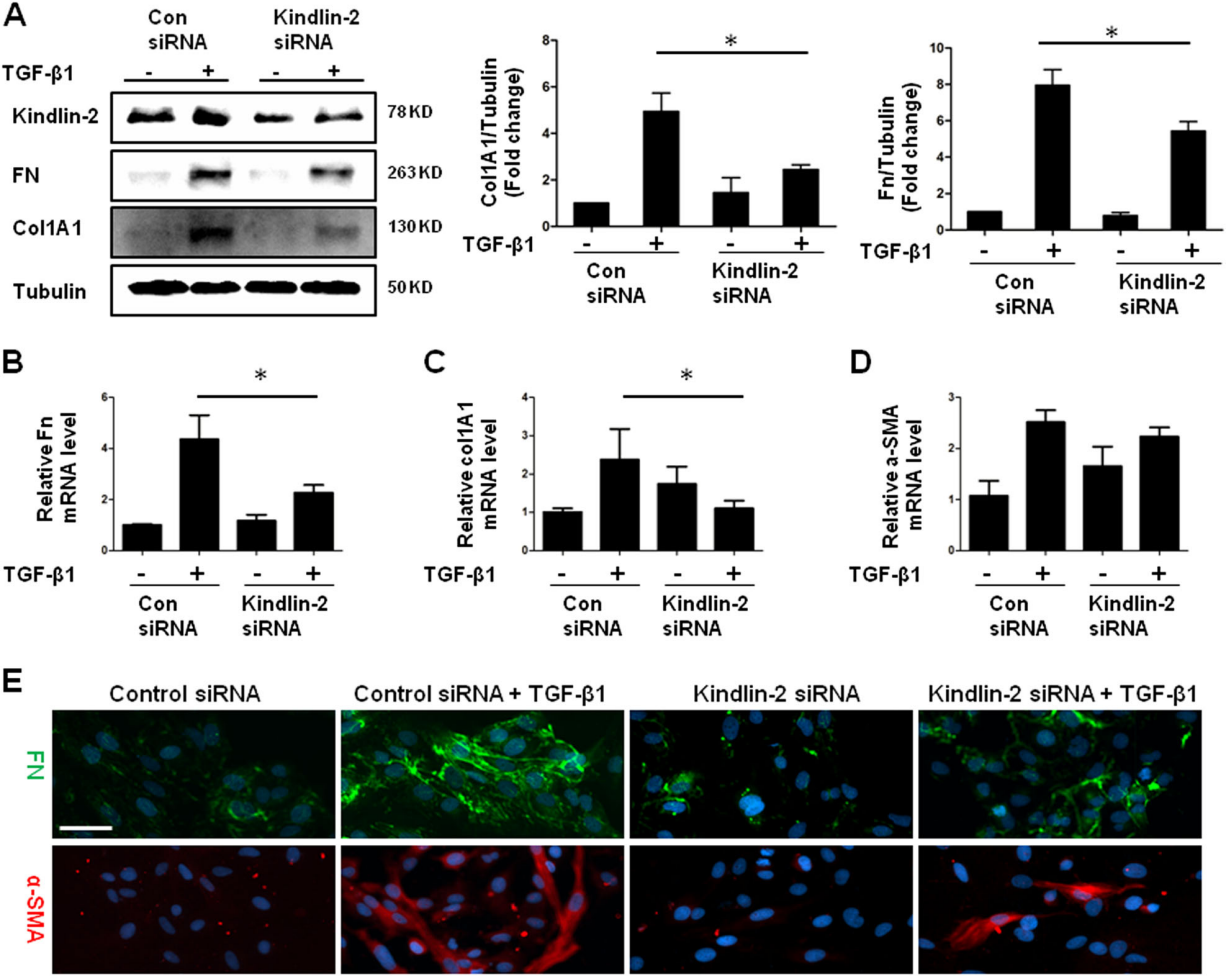
Inhibition of Kindlin-2 diminishes the pro-fibrogenic activities of TGF-β1. **a** LX-2 cells are transfected with control siRNA or Kindlin-2 siRNA followed by TGF-β1 (10 ng/ml) treatment for 48 h. The expressions of Kindlin-2, Col1A1, and Fn were determined by western blot. **b**–**d** After transfection with control siRNA or Kindlin-2 siRNA, LX-2 cells were treated with or without TGF-β1 (10 ng/ml) treatment for 24 h. Real-time PCR analyses of Fn, Col1A1, and α-SMA mRNA were performed. **e** Immunofluorescence analysis of Fn and α-SMA from LX-2 cells that were transfected with control siRNA or Kindlin-2 siRNA and then treated with or without 10 ng/ml TGF-β1 for 48 h. The data are representative of three independent experiments. Scale bars = 200 μm. *p < 0.05

**Fig. 4:**
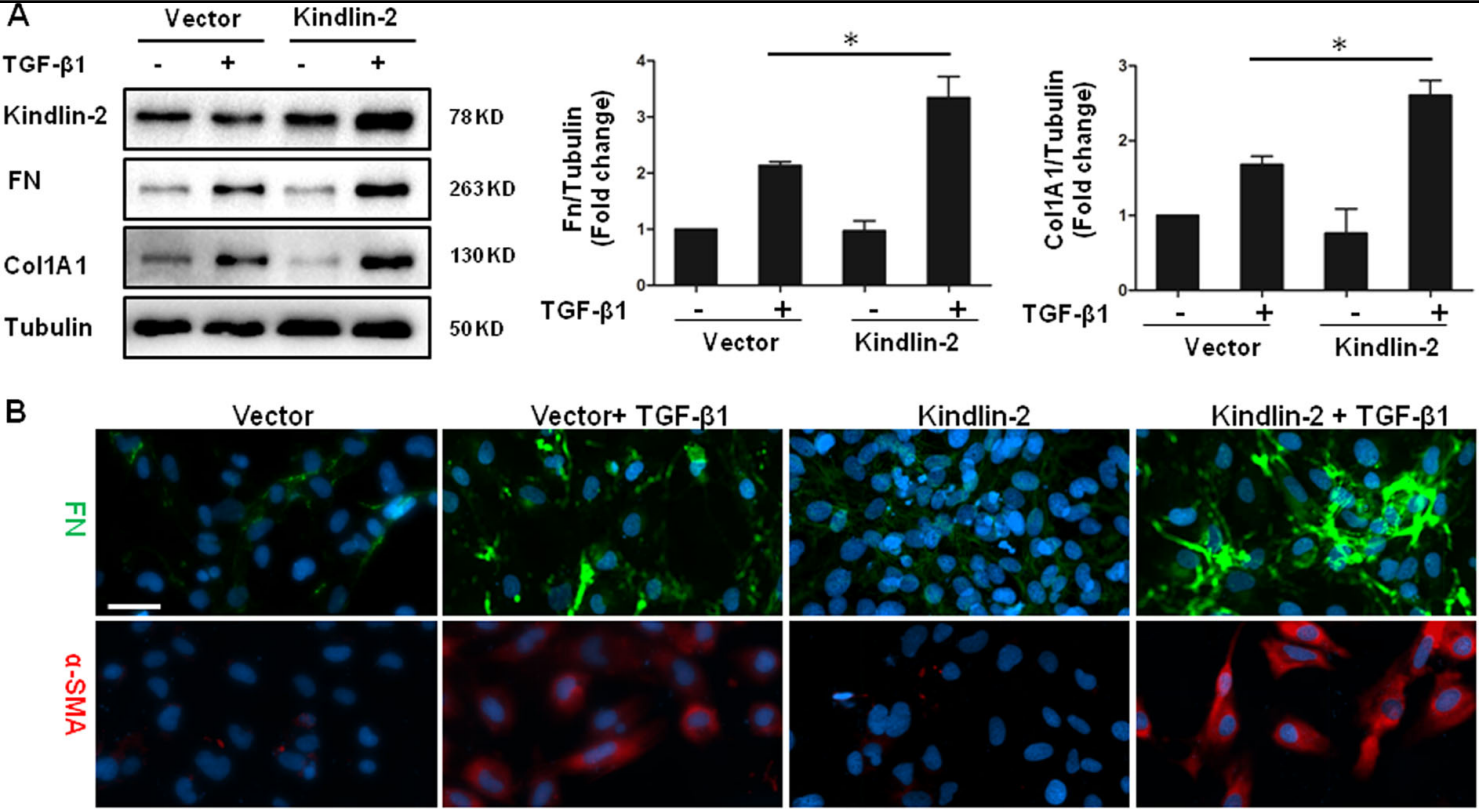
Overexpression of Kindlin-2 promotes TGF-β1-induced HSC activation. **a** LX-2 cells were transfected with control vector or Kindlin-2 vector followed by TGF-β1 (10 ng/ml) treatment for 48 h. The levels of Kindlin-2, Col1A1, and Fn were determined by western blot analysis. **b** LX-2 cells were transfected and treated as in **a**–**c**. Immunofluorescence assays were performed with anti-Fn and anti-α-SMA antibodies. The data are representative of three independent experiments. Scale bars = 200 μm. *p < 0.05

To investigate the functional effects of the increased expression of Kindlin-2, we used siRNA to knock down Kindlin-2 in LX-2 cells. Indeed, siRNA against Kindlin-2 successfully reduced the expression of Kindlin-2. Knockdown of Kindlin-2 dramatically blocked TGF-β-induced Col1A1 and FN protein expressions (Fig. 3a). Kindlin-2 silencing also significantly suppressed the mRNA levels of Col1A1, FN, and α-SMA, as showed in Fig. 3b–d. Furthermore, immunofluorescence staining demonstrated that knockdown of Kindlin-2 substantially attenuated the expressions of both FN and α-SMA protein levels stimulated by TGF-β1 (Fig. 3e). To further investigate the roles of Kindlin-2 in regulating HSC activation, we evaluated the overexpression of Kindlin-2 on LX-2 cells. As shown in Fig. 4a, enforced Kindlin-2 expression enhanced TGF-β-induced expressions of Col1A1 and FN. In addition, Kindlin-2 overexpression upregulated FN and α-SMA protein levels triggered by TGF-β1 that was confirmed by immunofluorescence assay (Fig. 4b).

### Kindlin-2 contributes to the activation of TGF-β/Smad pathway

**Fig. 5:**
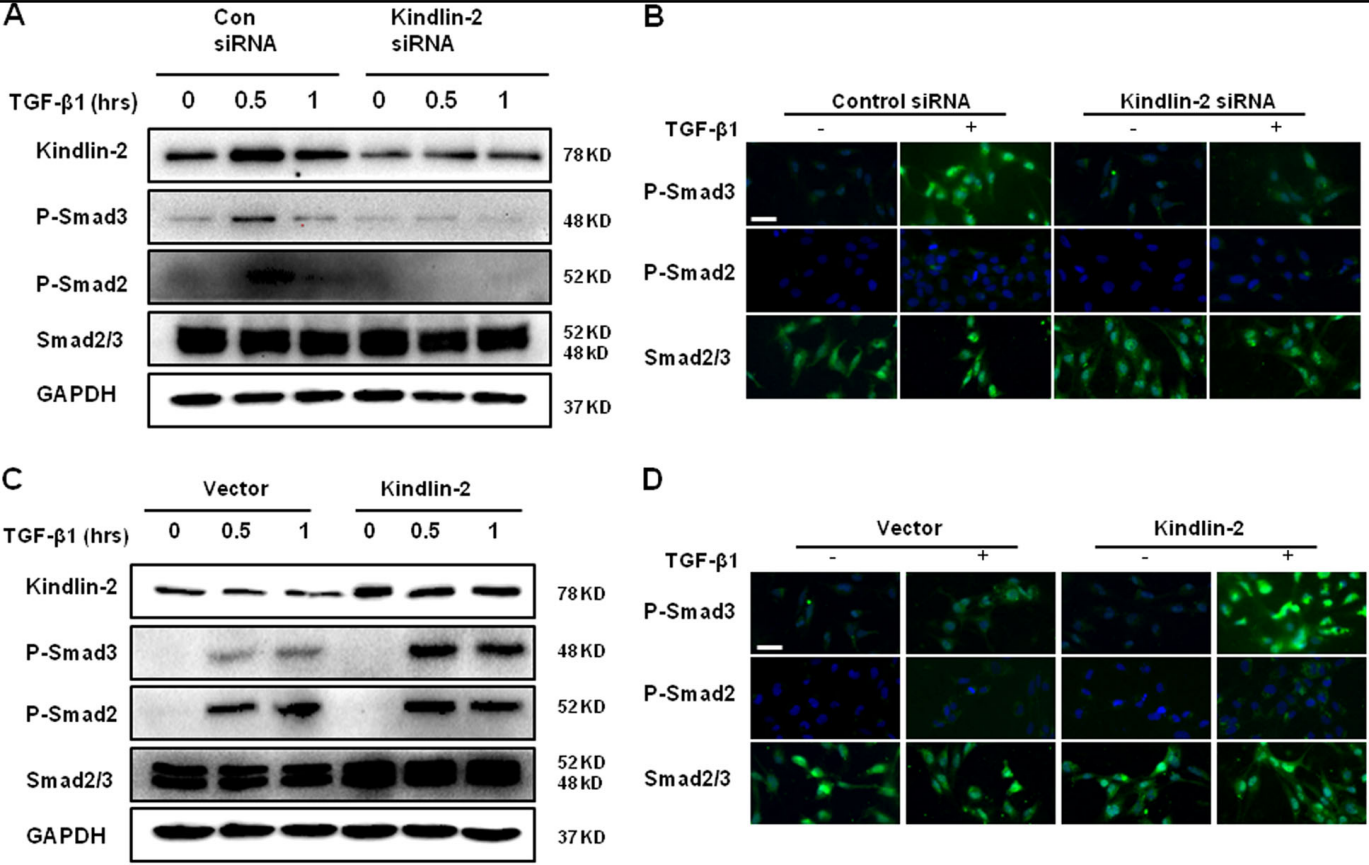
Kindlin-2 promotes Smad2/3 phosphorylation. **a** LX-2 cells transfected with control siRNA or Kindlin-2 siRNA and further treated with 10 ng/ml TGF-β1 for the indicated time period. P-Smad2/3 and total Smad2/3 levels were detected by western blot. **b** LX-2 cells transfected with control siRNA or Kindlin-2 siRNA and further treated with 10 ng/ml TGF-β1 for 30 min. The expressions of P-Smad2/3 and total Smad2/3 were determined by immunofluorescence assays. **c** LX-2 cells were transfected with control vector or Kindlin-2 vector, followed by TGF-β1 (10 ng/ml) treatment for the indicated time period. P-Smad2/3 and total Smad2/3 levels were detected by western blot. **d** LX-2 cells were transfected with control vector or Kindlin-2 vector, followed by TGF-β1 (10 ng/ml) treatment for 30 min. The expressions of P-Smad2/3 and total Smad2/3 were determined by immunofluorescence assays. The data are representative of three independent experiments. Scale bars = 200 μm

TGF-β/Smad signaling was demonstrated to participate in HSC activation and ECM production. Therefore, we determined that whether Kindlin-2 is required for the activation of TGF-β/Smad pathway. As shown in Fig. 5a, knockdown of Kindlin-2 with siRNA decreased phosphorylation of Smad2 and Smad3 protein levels in response to TGF-β1 compared with control siRNA. In addition, immunofluorescence staining showed similar effect (Fig. 5b). Consistently, overexpression of Kindlin-2 indeed enhanced the TGF-β1-induced phosphorylation of Smad2 and Smad3 (Fig. 5c), and these results were also observed using immunofluorescence assay (Fig. 5d). These results indicate that Kindlin-2 activates TGF-β/Smad signaling through upregulation of Smad2 and Smad3 phosphorylation.

### Kindlin-2 deficiency reduces CCl_4_-induced liver fibrosis

**Fig. 6:**
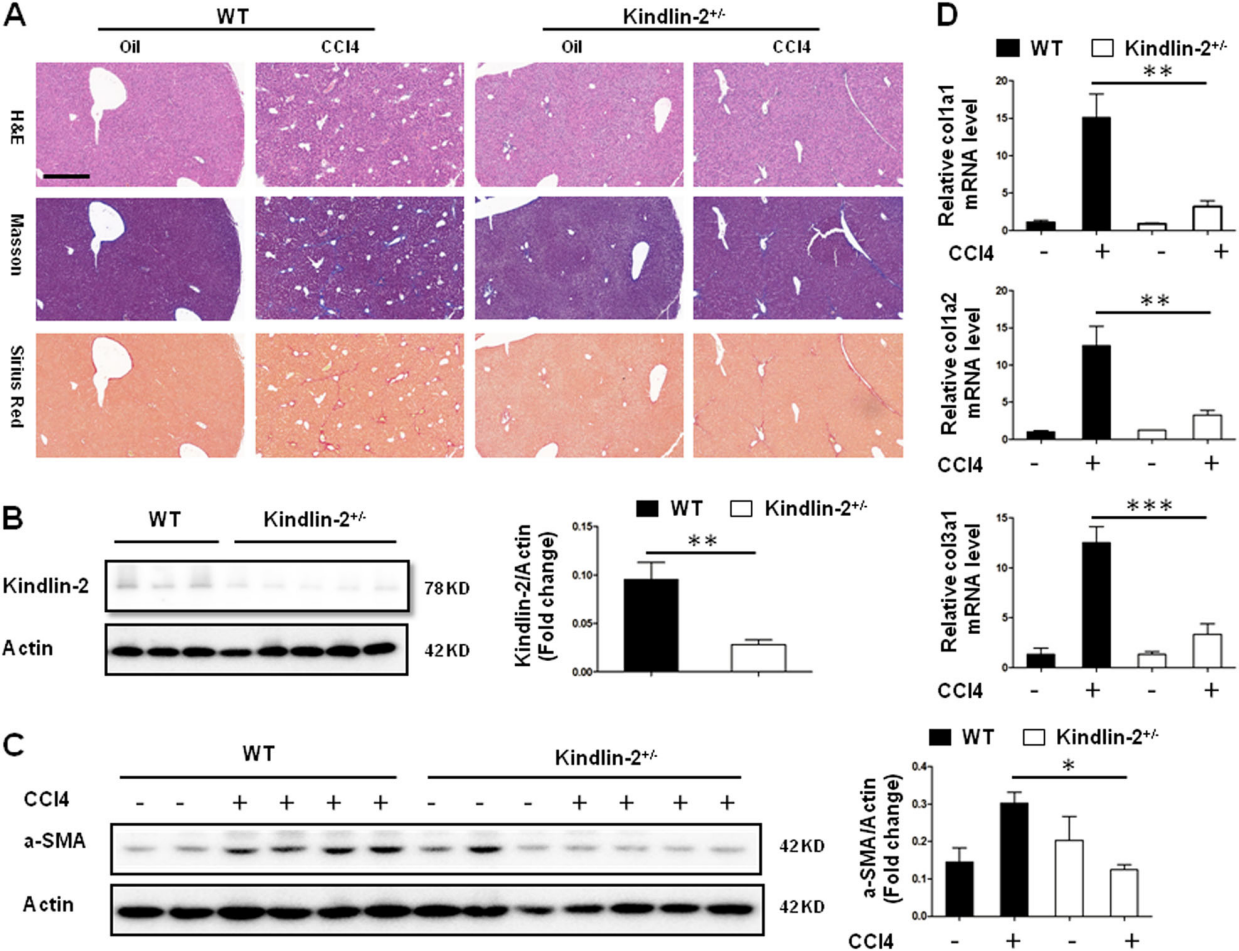
Kindlin-2 deficiency attenuates mouse liver fibrosis following CCl_4_ treatment. WT and Kindlin-2^+/−^ mice (*n* = 6–8 in each group) underwent CCl_4_ injection for 6 weeks. **a** Liver fibrosis was evaluated by H&E, Sirius Red, and Masson’s trichrome staining. Scale bars = 200 μm. **b** Western blot analysis of Kindlin-2 in livers from WT and Kindlin-2^+/−^ mice. The right panels show quantitative analyses of Kindlin-2 expression after normalization with β-actin. **c** Representative western blot analysis of α-SMA expressions in the livers from WT and Kindlin-2^+/−^ mice. The right panels show quantitative analyses of α-SMA expression after normalization with β-actin. **d** The mRNA expressions of col1a1, col1a2, and col3a1 were determined by real-time PCR. *p < 0.05, **p < 0.01, ***p < 0.001

To investigate the role of Kindlin-2 in experimental liver fibrosis, the mouse model of CCl_4_-induced liver fibrosis was used. As shown in Fig. 6b, the expression of Kindlin-2 protein in Kindlin-2^+/−^ mouse livers was significantly reduced compared with control mice. The expression of beta-actin was not changed after Kindlin-2 depletion. After the treatment of mice with CCl_4_, we found that Kindlin-2 depletion significantly attenuated the liver collagen deposition illustrated by Sirius Red and Masson’s trichrome staining (Fig. 6a). Moreover, we assessed the protein levels of α-SMA in the liver by western blot analysis. α-SMA protein levels were markedly diminished in the liver from CCl_4_-treated Kindlin-2^+/−^ mouse compared with WT mouse (Fig. 6c). Next, the mRNA expressions of Col1A1, Col1A2, and Col3A1 were significantly reduced in Kindlin-2^+/^^−^ mice livers compared with WT mouse after CCl_4_ injection (Fig. 6d). Collectively, these results demonstrated that Kindlin-2 deficiency significantly attenuated CCl_4_-induced liver fibrosis, suggesting that Kindlin-2 is a potential therapeutic target for liver fibrosis.

## Discussion

In this article, we demonstrated for the first time that Kindlin-2 plays a key role in HSC activation and liver fibrosis. First, we showed that Kindlin-2 expression was markedly upregulated in mouse and human fibrotic livers. Second, TGF-β1 significantly induced Kindlin-2 expression in vitro, and knockdown of Kindlin-2 suppressed TGF-β1-induced HSC activation. Third, Kindlin-2 promoted TGF-β signaling through activation of Smad2 and Smad3 phosphorylation. Finally, depletion of Kindlin-2 in vivo markedly attenuated CCl_4_-induced mouse liver fibrosis. All these data support our conclusion that Kindlin-2 functions as a positive regulator in liver fibrosis and may be a promising target for liver fibrosis treatment.

Recently, Lin et al. reported that Kindlin-2 expression is upregulated in hepatocellular carcinoma (HCC) and promotes HCC progression^26^; however, the function of Kindlin-2 in other liver diseases including liver fibrosis remains unknown. In the current study, we showed that Kindlin-2 expression was enhanced in livers from both human liver fibrosis patients and mouse liver fibrosis model. Moreover, Kindlin-2 mRNA and protein levels were elevated in a time-dependent manner in TGF-β1-stimulated LX-2 cells. This result was consistent with the recent reports that Kindlin-2 expression was increased in human fibrotic kidney and TGF-β1-treated human renal tubular epithelial cells (HKCs)^21^. In another study, Kindlin-2 was also increased significantly in pancreatic ductal adenocarcinoma (PDAC) cells stimulated with TGF-β1^27^. Nevertheless, little is known about the regulatory mechanism of increased Kindlin-2 expression induced by TGF-β1. In the current study, TGF-β1-induced Kindlin-2 expression was abrogated by p38 and ERK inhibitor, which indicated that Kindlin-2 expression may be regulated, at least in part, by noncanonical Smad pathway. However, the detailed mechanism of how TGF-β1 acts on the expression of Kindlin-2 needs further investigation.

HSC plays a pivotal role in the pathogenesis of liver fibrosis, and inhibition of HSC activation has been accepted as a major strategy to resolve liver fibrosis^28,29^. Accumulating studies uncovered various pro-fibrogenic pathways involved in liver fibrosis, including TGF-β/Smad, Wnt/β-catenin, and PDGF pathways^30^. Among them, TGF-β/Smad pathway is the most important in the progression of fibrotic diseases. Our study clearly demonstrated that knockdown of Kindlin-2 by siRNA dramatically reduced the TGF-β1-induced HSC activation. Furthermore, siRNA-mediated silencing of Kindlin-2 inhibited TGF-β1-induced Smad2/3 phosphorylation. These data suggest that Kindlin-2 is required for TGF-β1-mediated HSC activation. However, more experiments should be carried out to unravel the precise mechanism.

Several independent teams have reported paradoxical results about the role of Kindlin-2 in fibrotic diseases. Wei et al. demonstrated that blockade of Kindlin-2 in vivo ameliorated unilateral ureteral obstruction (UUO)-induced renal fibrosis^21^; on the contrary, postnatal loss of Kindlin-2 led to the enlargement of the heart and extensive fibrosis^22,23^. In this study, we clearly revealed that Kindlin-2^+/−^ mice were protected against CCl_4_-induced liver fibrosis, which is in line with our data of in vitro experiments. It is worth to note that we and other teams used different transgenic mice and fibrotic animal models. These findings indicate a distinct role of Kindlin-2 in different organ fibrogenesis. Thus, our study and theirs together provide strong evidence that Kindlin-2 plays a crucial role in fibrotic disorders, positive or negative. It will be interesting to evaluate the role of Kindlin-2 in other fibrotic diseases.

Liver fibrosis is a complex disorder that involves many cell types including HSC, hepatocytes, Kupffer cells, and infiltrated inflammatory cells^4^. Apart from activated HSC, hepatocytes derived from epithelial mesenchymal transition (EMT) have been revealed to be another important source of ECM^31^. Anyway, this study focuses on the effect of Kindlin-2 on HSC activation. It will be interesting to see if Kindlin-2 influences hepatocytes apoptosis and EMT during liver fibrosis. Although TGF-β1 is the most important pro-fibrogenic factor, accumulating studies have revealed multiple factors such as PDGF and Wnt are involved in the activation of HSCs and the pathogenesis of liver fibrosis^32,33^. Kindlin-2 acts as an adapter protein which forms protein complex with signaling components, then facilitates downstream signal transduction. Therefore, Kindlin-2 may likely participate in various intracellular signaling. In this study, we uncovered the role Kindlin-2 in TGF-β1 signaling; however, future studies should focus on the role of Kindlin-2 in other pro-fibrogenic signaling and the cross talk between multiple pathways.

In summary, our findings in the present study identified that Kindlin-2 may play a pivotal role in HSC activation and liver fibrosis progression by both in vitro and in vivo approaches. Kindlin-2 is regulated by TGF-β/Smad pathway and contributes to the phosphorylation of Smad2 and Smad3. Importantly, Kindlin-2 depletion attenuates liver fibrosis in a CCl_4_ mouse model of liver fibrosis. These results indicate that Kindlin-2 plays a key role in the process of liver fibrogenesis and represent a new therapeutic target to effectively limit the progression of liver fibrosis.

## Materials and methods

### Human liver samples

Human liver samples were obtained from patients undergoing liver resection at the Tongji Hospital. The study protocol was approved by the Institutional Review Board of Tongji Hospital, and informed consent in writing was obtained from each patient.

### Mice

Kindlin-2^+/−^ mice in the C57BL/6 background were purchased from the Model Animal Research Center of Nanjing University (Nanjing, China). Protocols for animal use were approved by the Institutional Animal Care and Use Committee (IACUC) at Tongji Hospital in accordance with the National Institutes of Health guidelines. Liver fibrosis was induced by intraperitoneal injection of carbon tetrachloride CCl_4_ (1:5 dilution in olive oil, 1 ml/kg body, three times per week for 6 weeks)^34^.

### Cell culture and treatment

Human HSC cell line, LX-2 cells, was cultured in Dulbecco’s modified Eagle’s medium (DMEM) containing 10% fetal bovine serum (FBS) and 1% penicillin/streptomycin, and maintained in a humidified incubator with 5% CO_2_ at 37 °C. LX-2 cells were serum-starved with 0.5% FBS for 24 h before intervention.

### Reagents and chemicals

Recombinant human TGF-β1 was purchased from Peprotech (Rocky Hill, NJ, USA), reconstituted in DMEM containing 10% FBS, and utilized at a final concentration of 10 ng/ml. JNK inhibitor SP600125, ERK inhibitor U0126, and p38 MAPK inhibitor SB203580 were purchased from SelleckChem (Houston, TX, USA), dissolved in DMSO, and utilized at a final concentration of 10 μM, 1 μM, and 1 μM, respectively.

### RNA interference analysis

siRNA targeting the Kindlin-2 sequences and non-targeting siRNA were synthesized by RiboBio Co., Ltd. (Guangzhou, China). The sense targeting sequence was as follows: AAGCUGGUGGAGAAACUCG^17^. LX-2 cells were transfected with Kindlin-2 or control siRNA at 50% confluence using Lipofectamine 2000 (Invitrogen) according to the manufacturer’s instructions.

### Overexpression of Kindlin-2

To force overexpression of Kindlin-2, pEGFP-Kindlin-2 (Vigene Biosciences, Shandong, China) was transfected into LX-2 cells using Lipofectamine 2000. Twenty-four hours after transfection, the cells were treated as indicated and assessed by western blot.

### Western blot analysis

Western blot analysis was performed as described previously^35^. The primary antibodies used in western blot were: Kindlin-2 (Proteintech, 1:1000 dilution), Fibronectin(FN) (Proteintech, 1:1000 dilution), Col1A1 (Boster, 1:400 dilution), α-SMA (Abcam, 1:100 dilution), Smad2/3 (Cell Signaling Technology, 1:1000 dilution), p-Smad2 (Cell Signaling Technology, 1:1000 dilution), p-Smad3 (Cell Signaling Technology, 1:1000 dilution), GAPDH (Abacm, 1:3000 dilution), β-actin (Abacm, 1:3000 dilution), and tubulin (Abcam, 1:3000 dilution). Detection was performed using a chemiluminescent substrate system (Bio-Rad). The gray values were analyzed with ImageJ software.

### Quantitative real-time polymerase chain reaction

Total RNA from liver tissues or LX-2 cells was extracted by Trizol (Takara, Dalian, China). The single-stranded cDNA was synthesized from total RNA using PrimeScript^TM^ RT reagent kit (Takara). Real-time PCR was set up using SYBR Green mix (Takara) under the following condition: 30 s at 95 °C for initial denaturation, followed by 40 cycles of 95 °C for 5 s and 60 °C for 30 s on the Applied Biosystems 7500 Real-Time PCR system (Thermo Fisher Scientific, USA). The levels of β-actin RNA expression were used to normalize the data. The sequences of primers for real-time PCR are listed in Supplementary Table 1.

### Histological analysis of liver sections

The specimens were fixed in 4% paraformalin and embedded in paraffin in preparation for histopathological analysis. Sections were stained with hematoxylin and eosin (H&E) for morphology, and Masson’s trichrome and Sirius red for fibrosis.

### Immunofluorescence staining

Immunofluorescence staining was performed as described previously^36^. Briefly, cells cultured on the glass coverslips or tissue sections were stained with following antibodies: Kindlin-2 (Proteintech, 1:100 dilution), Fibronectin (FN) (Abcam, 1:100 dilution), α-SMA (Abcam, 1:100 dilution), Smad2/3 (Cell Signaling Technology, 1:100 dilution), p-Smad2 (Cell Signaling Technology, 1:100 dilution), and p-Smad3 (Cell Signaling Technology, 1:100 dilution) overnight at 4 °C, followed by incubation with FITC or PE-conjugated secondary antibodies (Invitrogen). The nuclei were the stained with DAPI. All immunofluorescence was then visualized by Carl Zeiss MicroImaging system (Carl Zeiss, Germany).

### Statistical analysis

Data were expressed as mean ± SEM. Differences between two groups were examined for statistical significance using the Student’s *t* test. For comparisons between multiple groups, one-way ANOVA followed by Tukey’s test was performed. *P* < 0.05 was considered significant.

## Electronic supplementary material

Supplementary Table 1
