# Supplementary material for: Kindlin-2 regulates hepatic stellate cells activation and liver fibrogenesis
Source: Cell Death Discov. 2018 Sep 12;4:93. doi: 10.1038/s41420-018-0095-9 (PMC6135746; doi:10.1038/s41420-018-0095-9)
Supplement: Supplementary file 1 — Supplementary Table 1 [file 41420_2018_95_MOESM1_ESM.doc]

| **Species** | **Gene** | **Forward primer** | **Reserve primer** |
| --- | --- | --- | --- |
| **Human** | beta-actin | GCAAGCAGGACTATGACGAG | CAAATAAAGCCATGCCAATC |
|  | a-SMA | GGCATTCACGAGACCACCTAC | CGACATGACGTTGTTGGCATAC |
|  | Col1a | TTCTTGCAGTGGTAGGTGATGTTC | GCTACCCAACTTGCCTTCATG |
|  | Fn | GTGTTGGGAATGGTCGTGGGGAATG | CCAATGCCACGGCCATAGCAGTAGC |
| **Mouse** | beta-actin | GGCTGTATTCCCCTCCATCG | CCAGTTGGTAACAATGCCATGT |
|  | a-SMA | CCACCGCAAATGCTTCTAAGT | GGCAGGAATGATTTGGAAAGG |
|  | Col1a1 | CAAGAAGACATCCCTGAAGTC | ACAGTCCAGTTCTTCATTGC |
|  | Col1a2 | ACCCGATGGCAACAATGGA | ACCAGCAGGGCCTTGTTCAC |
|  | Col3a1 | AACCTGGTTTCTTCTCACCCTTC | ACTCATAGGACTGACCAAGGTGG |
|  | Fn | TCTGGGAAATGGAAAAGGGGAATGG | CACTGAAGCAGGTTTCCTCGGTTGT |
